# Frequent Medical Supervision Increases the Effectiveness of a Longitudinal Multidisciplinary Body Weight Reduction Program: A Real-World Experience in a Population of Children and Adolescents with Obesity

**DOI:** 10.3390/nu13103362

**Published:** 2021-09-25

**Authors:** Antonello E. Rigamonti, Diana Caroli, Graziano Grugni, Silvano G. Cella, Alessandro Sartorio

**Affiliations:** 1 Department of Clinical Sciences and Community Health, University of Milan, 20129 Milan, Italy; silvano.cella@unimi.it; 2 Experimental Laboratory for Auxo-Endocrinological Research, Istituto Auxologico Italiano, IRCCS, 28824 Verbania, Italy; d.caroli@auxologico.it (D.C.); g.grugni@auxologico.it (G.G.); sartorio@auxologico.it (A.S.); 3 Division of Auxology and Metabolic Diseases, Istituto Auxologico Italiano, IRCCS, 28824 Verbania, Italy

**Keywords:** obesity, medical supervision, children, adolescents, body weight reduction program, metabolic syndrome, real-world evidence

## Abstract

Regular medical supervision represents a fundamental component of the clinical management of obesity. In fact, when frequently supplied it reduces the risk of failure associated with any body weight reduction program (BWRP), resulting in body weight gain. The aim of the present study was to establish the potential beneficial effects of increasing medical supervision on weight loss and other auxometric and cardiometabolic parameters in a population of children and adolescents with obesity (*n* = 158; F/M = 94/64; age range 9.7–17.3 years; body mass index, BMI = 37.8 ± 6.9 kg/m^2^), followed up for one year in a real-world setting, after and before a 3-week in-hospital BWRP. Weight loss was significantly associated with medical supervision and changes in several auxometric and cardiometabolic parameters such as fat mass, fat-free mass, waist and hip circumferences, total and LDL cholesterols, triglycerides, glucose, insulin, HOMA-IR, systolic blood pressure and IDF criteria for the diagnosis of metabolic syndrome. As expected, weight loss and, congruently, medical supervision, were significantly higher in responsive and stable subjects than in those belonging to the non-responsive group and in responsive subjects than those belonging to the stable group. While weight loss was significantly higher in subjects having class 2 and 3 obesity than those belonging to class 1 obesity group, medical supervision was significantly higher in subjects having class 3 than those having class 1 obesity. Weight loss was significantly higher in subjects suffering from metabolic syndrome than those without; nevertheless, no significant difference was found in medical supervision between these groups. Finally, sex was associated with no differences in weight loss and medical supervision. In conclusion, based on the results of a real-world experience, frequent medical supervision increases the weight loss associated with a longitudinal multidisciplinary BWRP, with a parallel improvement of a set of auxometric and cardiometabolic parameters. Prospectively, incentivising regular medical supervision should reduce the risk of BWRP failure and body weight gain, thus contributing to counteract the detrimental transition from simple obesity to metabolic syndrome in pediatric patients.

## 1. Introduction

Over the past two decades, pediatric obesity has become a major public health burden not only in Western countries but widespread all over the world, such that the World Health Organisation (WHO) has defined obesity as a global epidemic of the third millennium [1]. Treatment options for reducing body weight include dietary intervention, physical activity, behavioural modification, pharmacotherapy and surgery, although the last two are less frequently adopted in pediatric patients [2]. However, the complexity of obesity, which is fundamentally a chronic condition, and the medical importance of preventing an obese child/adolescent from becoming an obese adult implies adoption of an approach based on an integrated multidisciplinary team to the care of obese patients, particularly those who, due to different reasons, fail weight control [3].

The long-term duration of the treatment, such as body weight reduction programs (BWRPs), and the need for monitoring patients’ adherence and interventions effectiveness, should be considered as a clinical challenge and not as an unavoidable limitation [4].

Different medical strategies have been proposed for maintenance of an initially effective BWRP in overweight and obese patients [5]. In particular, the following interventions have been reported to be the most effective: having healthy foods available at home, regular breakfast intake, increasing vegetable consumption, decreasing sugary foods, and limiting fat in meals. Furthermore, increased physical activity has been recognised as the most consistent positive correlate of weight loss maintenance. Finally, psychological counselling, together with nutritional education, represents a valuable strategy to improve patient’s adherence [6].

In this regard, medical supervision, i.e., in- and out-patient healthcare services specifically devoted to the treatment of obese patients, appears to be fundamental not only for adjusting and, consequently, tailoring the treatment(s) over a long time, particularly when comorbidities are present such as metabolic syndrome, but also for psychologically motivating the obese patient, who, discouraged due to meager results, might drop out from the treatment [4,7]. Because the pediatric obese population are particularly vulnerable, with a high rate of body weight gain after an initially effective BWRP [8], medical supervision should be at a patient’s request rather than a physician’s recommendation/choice.

Although the importance of medical supervision may be an apparently obvious concept, valid not only for obesity, but also for other chronic diseases such as blood hypertension [9] or diabetes mellitus [10], to the best of our knowledge, the real-world evidence of the association of a pressing medical supervision with a progressively increasing weight loss after an initially effective BWRP has only been partially investigated.

Therefore, the aim of the present study was that of quantitatively establishing the potential beneficial effects of an increasing medical supervision on weight loss and other auxometric and cardiometabolic parameters in a population of children and adolescents with obesity, followed for one year in a real-world setting, after and before a 3-week in-hospital BWRP. Our hypothesis is that even one intervention of medical supervision is capable of further increasing weight loss and improving other auxometric and cardiometabolic parameters.

## 2. Materials and Methods

### 2.1. Subjects

A set of 162 children and adolescents was selected from a patient population admitted to the Division of Auxology of the Istituto Auxologico Italiano, Piancavallo (VB), Italy, between January 2016 and January 2018 for a 3-week in-hospital multidisciplinary BWRP.

The inclusion criteria were individuals of both sexes, aged between 5 and 18 years, having a body mass index (BMI) (or BMI deviation standard score, BMI-SDS) >95th percentile from age- and sex-specific Italian charts [11], with or without metabolic syndrome (see below for its definition), independent of the level of physical activity. The exclusion criteria were: (1) secondary causes of obesity (e.g., Prader–Willi syndrome, steroid-induced or medication-induced obesity); (2) individuals with systolic blood pressure (SBP) ≥180 mmHg and diastolic blood pressure (DBP) ≥110 mmHg; (3) cardiovascular disease clinically evident in the previous 6 months; (4) psychiatric, neurological, osteomuscular, or rheumatologic diseases that limit the ability to undertake a (standard) 3-week in-hospital period of metabolic rehabilitation, including physical activity (see below for details); (5) individuals (and/or their parents) who refused to sign the consent form.

After clinical evaluation, based on the above-reported inclusion/exclusion criteria, four patients were excluded from the study due to a spontaneous withdrawal for personal reasons (no. 3, i.e., early discharge occurring after less than 10 days from the beginning of the 3-week BWRP) and young age (no. 1, i.e., 5-year-old), thus leading to a total of 158 patients to be recruited and included in the statistical analysis (94 females and 64 males, age range 9.7–17.3 year; BMI = 37.8 ± 6.9 kg/m^2^; BMI-SDS = 3.0 ± 0.6). Of these, 25 (15.8%) were taking medications for diabetes (metformin: no. 24, insulin: no. 1), 2 (1.3%) for hypertension, and 2 (1.3%) for dyslipidemia.

The protocol was approved by the Ethical Committee of the Istituto Auxologico Italiano (research project code: 01C504; acronym: LITOB); the protocol was explained to the patients and/or their parents, who gave their written informed consent.

During the 1-year period intercurrent between two consecutive 3-week BWRPs (at T1 and T2), in a real-world setting, each subject could (or not) undergo a medical supervision, consisting of (cost-free, i.e., covered by the National Health System) out-patient clinic or day-hospital with healthcare personnel having qualified expertise in the clinical management of obesity (e.g., endocrinologist, nutritionist, dietician, therapist, psychologist etc.) or hospitalisation (e.g., a third or more 3-week BWRP). The medical supervision included a general clinical evaluation, anthropometric measurements, nutritional and physical activity recordings, assessment of therapy compliance and, if necessary, adaptation of drugs posology.

The medical supervision was recommended by our medical or paramedical team, but each subject was free to adhere or not.

### 2.2. Body Weight Reduction Program (BWRP)

The BWRP consisted of a 3-week in-hospital integrated energy-restricted diet (1200–1800 kcal/day) in combination with physical rehabilitation (moderate aerobic activity), psychological counseling, and nutritional education. The amount of energy to be given with the diet was calculated by subtracting approximately 500 kcal from the measurement of resting energy expenditure, which was determined after an overnight fast by means of open-circuit, indirect computerised calorimetry (Vmax 29, Sensor Medics, Yorba Linda, CA, USA) with a rigid, transparent, ventilated canopy. The diet, in terms of macronutrients, contained 21% proteins, 53% carbohydrates, and 26% lipids; the daily estimated water content was 1000 mL, while the estimated salt content was 1560 mg Na^+^, 3600 mg K^+^, and 900 mg Ca^2+^. Extra water intake of at least 2000 mL/day was encouraged.

The physical activity program consisted of 5 days per week training, including (i) 1 h dynamic aerobic standing and floor exercise with arms and legs, at moderate intensity and under the guidance of a therapist; and (ii) either 20–30 min cycloergometer exercise at 60 W, or 3–4 km outdoor walking on flat terrain, according to individual capabilities and clinical status.

The subjects also underwent a psychological counseling program consisting of two or three sessions per week of individual and/or group psychotherapy performed by clinical psychologists. Furthermore, lectures, demonstrations, and group discussions, with or without a supervisor, took place daily.

### 2.3. Anthropometric Measurements

A scale with a stadiometer was used to determine height and weight (Wunder Sa.Bi., WU150, Trezzo sull’Adda, Italy). Waist circumference (WC) was measured with a flexible tape in standing position, halfway between the inferior margin of the ribs and the superior border of the crista, while hip circumference (HC) was measured at the largest parts around the buttocks. Body composition was measured by bioimpedance analysis (Human-IM Scan, DS-Medigroup, Milan, Italy) after 20 min of supine resting and in accordance with the international guidelines [12]. BMI, fat mass (FM) and fat-free mass (FFM) were determined in all subjects.

### 2.4. Metabolic Variables

Blood samples (about 10 mL) were collected at around 8:00 a.m. after an overnight fast at the beginning of the BWRP at T1 and T2. Total cholesterol (T-C), high-density lipoprotein cholesterol (HDL-C), low-density lipoprotein cholesterol (LDL-C), triglycerides (TG), glucose and insulin were measured.

Colorimetric enzymatic-assays (Roche Diagnostics, Monza, Italy) were used to determine serum T-C, LDL-C, HDL-C and TG levels. The sensitivities of the assays were 3.86 mg/dL [1 mg/dL = 0.03 mmol/L], 3.87 mg/dL [1 mg/dL = 0.03 mmol/L], 3.09 mg/dL [1 mg/dL = 0.03 mmol/L] and 8.85 mg/dL [1 mg/dL = 0.01 mmol/L], respectively.

Serum glucose level was measured by the glucose oxidase enzymatic method (Roche Diagnostics, Monza, Italy). The sensitivity of the method was 2 mg/dL [1 mg/dL = 0.06 mmol/L].

Serum insulin concentration was determined by a chemiluminescent immunometric assay, using a commercial kit (Elecsys Insulin, Roche Diagnostics, Monza, Italy). The sensitivity of the method was 0.2 µIU/mL [1 µU/mL = 7.18 pmol/L].

The intra- and inter-assay coefficients of variation (CVs) were the following: 1.1% and 1.6% for T-C, 1.2% and 2.5% for LDL-C, 1.8% and 2.2% for HDL-C, 1.1% and 2.0% for TG, 1.0% and 1.3% for glucose, and 1.5% and 4.9% for insulin.

### 2.5. Evaluation of Blood Pressure

Blood pressure was measured on the right arm, using a sphygmomanometer with appropriate pediatric cuff size, with the subject in a seated position and relaxed condition. The procedure was repeated three times at 10 min intervals; the means of the three values for SBP and DBP were recorded.

### 2.6. Definition of Metabolic Syndrome

According to the IDF (International Diabetes Federation) criteria for diagnosis of metabolic syndrome in children and adolescents [13], our patients were considered positive for the presence of metabolic syndrome if they had abdominal obesity (WC ≥ 90th percentile [14] for ages <16 years, and ≥94 cm for males and ≥80 cm for female for ages >16 years) plus two or more of the following factors: (i) increased TG level: ≥150 mg/dL (1.7 mmol/L) for ages <16 years and the same cut-off or specific treatment for this lipid abnormality for ages >16 years, (ii) reduced HDL-C: <40 mg/dL (1.03 mmol/L) for males and females for ages <16 years, and <40 mg/dL for males and <50 mg/dL (1.29 mmol/L) for females, or specific treatment for this lipid abnormality for ages >16 years, (iii) increased BP: SBP ≥ 130 mmHg or DBP ≥ 85 mmHg for ages <16 years, and same cut-off or treatment of previously diagnosed hypertension for ages >16 years, (iv) increased fasting glucose concentration ≥100 mg/dL (5.6 mmol/L) or previously diagnosed type 2 diabetes mellitus for all ages.

### 2.7. Statistical Analysis

The Sigma Stat 3.5 statistical software package (Systat Software, San Jose, CA, USA) was used for data analyses and GraphPad Prisma 5.0 software (GraphPad Software, San Diego, CA, USA) for data plotting.

Results are reported as mean ± SD (standard deviation) or percentage (as specified in tables).

Before applying any parametric test, normal distribution and linearity of each variable were verified. Alternatively, a non-parametric test was applied.

Linear regression (for all data) was used to evaluate the association of weight loss (ΔBMI or ΔBMI-SDS, i.e., [BMI_T2_–BMI_T1_] or [BMI-SDS_T2_–BMI-SDS_T1_]) as dependent variable with medical supervision (i.e., number of any kind of medical visit occurred in-between the BWRPs at T1 and T2) as independent variable.

Linear regression (for all data) was also used to confirm the beneficial effects of weight loss (i.e., ΔBMI as above defined, independent variable) on changes in auxometric and cardiometabolic parameters (ΔY, i.e., [Y_T2_–Y_T1_], dependent variable), such as FM, FFM, WC, HC, WHR (waist to hip ratio), T-C, HDL-C, LDL-C, TG, glucose, insulin, HOMA-IR (homeostatic model assessment for insulin resistance, calculated by using the formula {glucose [mmol/L] × insulin [mIU/L]/22.5}), SBP/DBP and IDF criteria for the diagnosis of metabolic syndrome.

Changes in the auxometric and cardiometabolic parameters, including medical supervision (as defined above), were compared among/between the following groups:

(1) responsive (ΔBMI, i.e., [BMI_T2_–BMI_T1_] < −5 kg/m^2^), stable (>−5 and <5 kg/m^2^) and non-responsive (>5 kg/m^2^) subjects;
(2) class 1 obesity (BMI_T1_ ≥ 95th percentile to <120% of 95th percentile for age and sex), class 2 obesity (BMI_T1_ ≥ 120% to <140% of 95th percentile or BMI ≥ 35 kg/m^2^) and class 3 obesity (BMI_T1_ ≥ 140% of 95th percentile or BMI ≥ 40 kg/m^2^) [15];
(3) with and without metabolic syndrome;
(4) females and males.

To compare three groups, a one-way ANOVA, followed by the post-hoc Boneferroni test, or one-way ANOVA on ranks, followed by the post-hoc Dunn test, was applied, when appropriate. To compare two groups, a Student *t*-test for unpaired data was applied.

Drugs prescription and prevalence of metabolic syndrome (at T2 vs. T1) were analyzed by using Fisher’s exact test.

A level of significance of *p* < 0.05 was used for all data analyses.

## 3. Results

### 3.1. Relationship of Weight Loss with Medical Supervision

**Figure 1:**
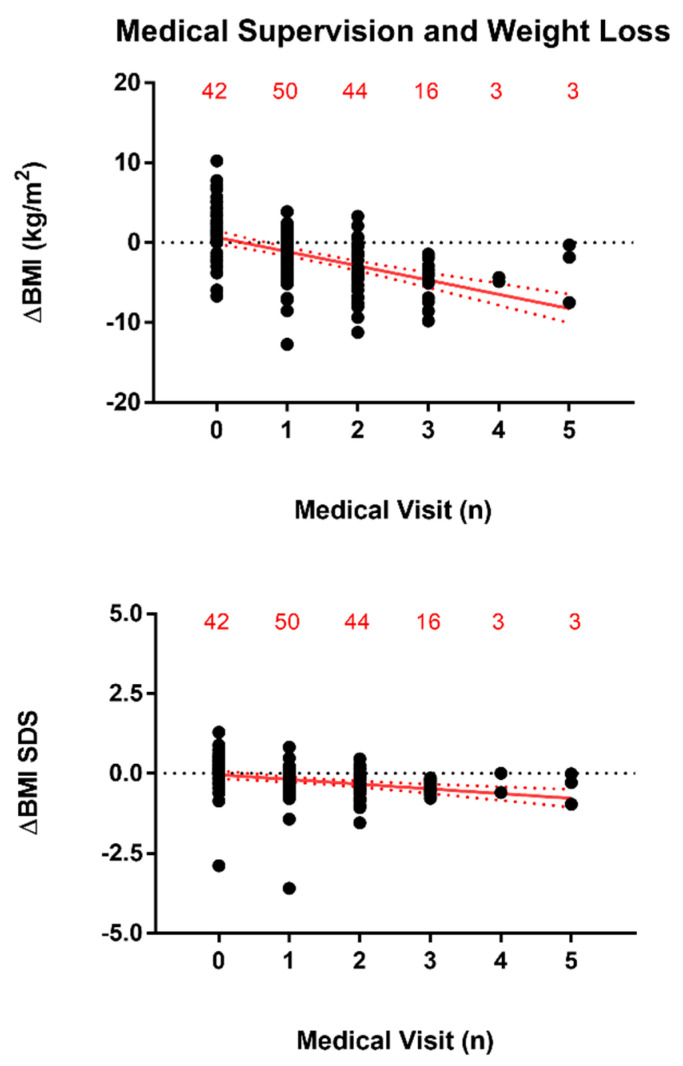
Linear regression (for all data, mean ± 95% confidence interval, CI) of weight loss (ΔBMI–top panel–or ΔBMI-SDS–bottom panel–, i.e., [BMI_T2_–BMI_T1_] or [BMI-SDS_T2_–BMI-SDS_T1_]) as dependent variable (y) with medical supervision (i.e., number of any kind of medical visit occurred in-between the BWRPs at T1 and T2) as independent variable (x). For ΔBMI: y = −1.785 × x + 0.6801 (r^2^ = 0.2678 and *p* < 0.0001). For ΔBMI SDS: y = −0.1476 × x–0.04101 (r^2^ = 0.09292 and *p* < 0.0001). Note: the number of obese subjects for each set of medical visits (0–5) are reported at the top of the two panels.

When considering all data, weight loss (ΔBMI or ΔBMI-SDS as a dependent variable [y], i.e., [BMI_T2_–BMI_T1_] or [BMI-SDS_T2_–BMI-SDS_T1_]) was significantly associated with medical supervision (i.e., number of any kind of clinical visit occurred in-between the BWRPs at T1 and T2 as independent variable [x]). In particular, the equation of linear regression for ΔBMI was the following: y = −1.785 × x + 0.6801 (r^2^ = 0.2678 and *p* < 0.0001), being that for ΔBMI-SDS the following: y = −0.1476 × x − 0.04101 (r^2^ = 0.09292 and *p* < 0.0001). The number of subjects for each set of medical visits were the following: 42 subjects without medical visits, 50 subjects received 1 medical visit, 44 subjects received 2 medical visits, 16 subjects received 3 medical visits, and 3 subjects received 4 and 5 medical visits (Figure 1).

### 3.2. Association of Weight Loss with Auxometric and Cardiometabolic Parameters

Table 1 reports the associations of weight loss (i.e., ΔBMI, the independent variable as defined above) with changes in auxometric and cardiometabolic parameters (ΔY, i.e., [Y_T2_–Y_T1_], dependent variable), confirming significant benefits (at least *p* < 0.05) on FM, FFM, WC, HC, T-C, LDL-C, TG, glucose, insulin, HOMA-IR, SBP and IDF criteria for the diagnosis of metabolic syndrome, with the exceptions of WHR, DBP and HDL-C.

Pharmaco-therapeutically, there were no significant differences in the drugs prescription (T2 vs. T1): in fact, one year after, 15 subjects (9.5%) were taking medication for diabetes (metformin), 4 (2.5%) for hypertension, and 4 (2.5%) for dyslipidemia.

### 3.3. Weight Loss and Medical Supervision in Specific Subgroups of Obese Subjects

#### 3.3.1. Response to BWRP

**Figure 2:**
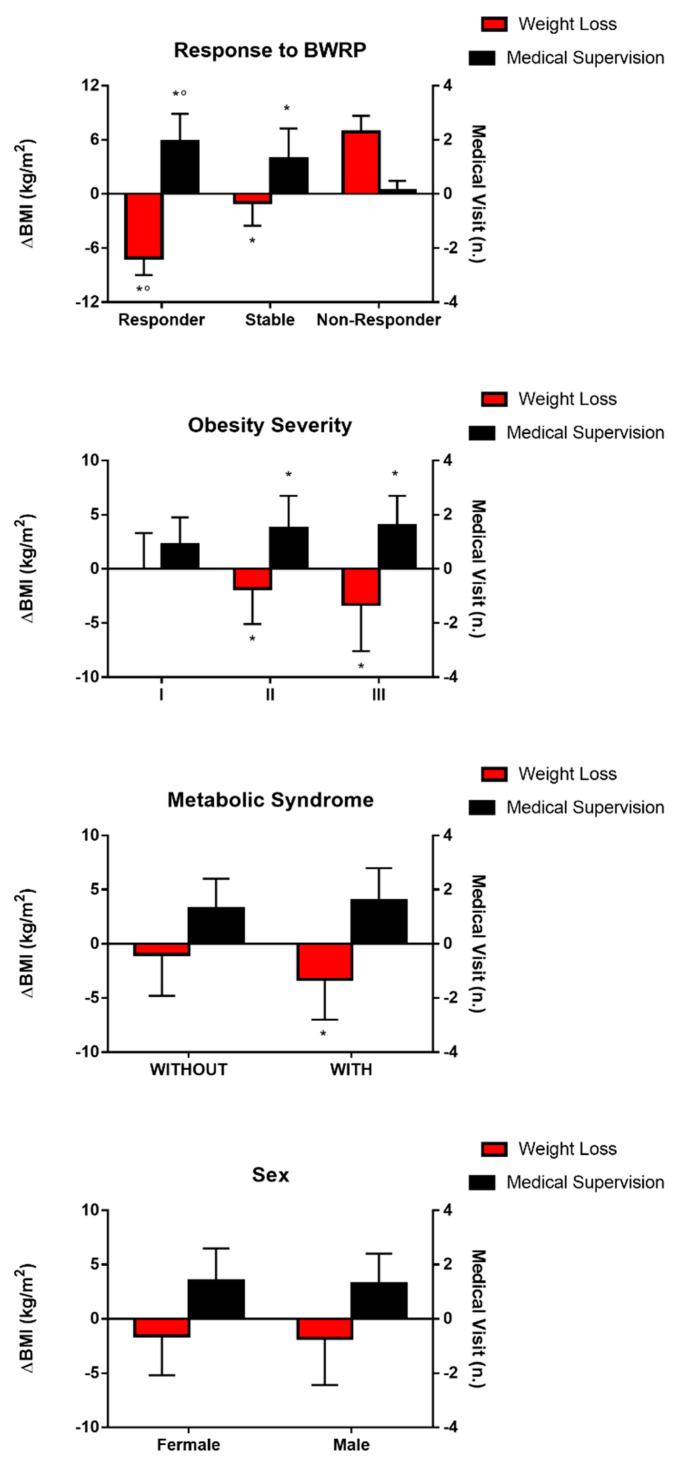
Weight loss (ΔBMI, i.e., [BMI_T2_–BMI_T1_]) and medical supervision (i.e., number of any kind of medical visit occurred in-between the BWRPs at T1 and T2) in four different subdivisions of the population of children/adolescents with obesity: (1) responsive (ΔBMI, i.e., [BMI_T2_–BMI_T1_] < −5 kg/m^2^), stable (>−5 and <5 kg/m^2^) and non-responsive (>5 kg/m^2^) subjects (first panel); (2) class 1 obesity (BMI_T1_ ≥ 95th percentile to <120% of 95th percentile for age and sex), class 2 obesity (BMI_T1_ ≥ 120% to <140% of 95th percentile or BMI ≥ 35 kg/m^2^) and class 3 obesity (BMI_T1_ ≥ 140% of 95th percentile or BMI ≥ 40 kg/m^2^) (second panel); (3) with and without metabolic syndrome (third panel); (4) females and males (fourth panel). * *p* < 0.05 vs. non-responsive subjects or class 1 obesity or without metabolic syndrome; ° *p* < 0.05 vs. stable subjects.

When subdividing our population of children and adolescents with obesity on the basis of the response to the BWRP at T1 (i.e., responsive, stable and non-responsive groups), as expected, ΔBMI was significantly higher in responsive and stable subjects than those belonging to the non-responsive group and in responsive subjects than those belonging to the stable group. These findings were congruent with medical supervision, which was significantly higher in responsive and stable subjects than those belonging to the non-responsive group and in responsive subjects than in those belonging to the stable group (Figure 2).

As reported in Table 2, the beneficial effects of the more frequent medical supervision and, consequently, of the more relevant weight loss in the responsive group were demonstrated by significant changes in auxometric and cardiometabolic parameters such as WC, HC, FM, FFM, HDL-C, LDL-C, TG, glucose, insulin, HOMA-IR and IDF criteria for the diagnosis of metabolic syndrome when compared with the stable and non-responsive groups (*p* < 0.05). These findings were less evident in the stable group when compared to the non-responsive one.

#### 3.3.2. Obesity Severity

While weight loss was significantly higher in subjects having class 2 and 3 obesity than those belonging to class 1 obesity (*p* < 0.05), medical supervision was significantly higher in subjects having class 3 obesity than those belonging to class 1 obesity (*p* < 0.05) (Figure 2).

#### 3.3.3. Metabolic Syndrome

Although not significant, a decrease in number of subjects having metabolic syndrome was found (T2 vs. T1: 40 [25.3%] vs. 48 [30.4%]).

Weight loss was significantly higher in subjects suffering from metabolic syndrome than those without (*p* < 0.05). Nevertheless, no significant difference was found in medical supervision between these groups (Figure 2).

#### 3.3.4. Sex

Finally, sex was associated with no differences in weight loss and medical supervision (Figure 2).

## 4. Discussion

The present report summarises a real-world experience carried out in a population of children and adolescents with obesity, who had undergone an initial 3-week BWRP and followed for one year, during which a physician’s recommended and/or patient’s requested medical supervision could occur at different frequency. The total number of access to in- and out-patient healthcare services ranged from 0 to 5 per year. Counselling by medical and paramedical personnel having qualified expertise in the clinical management of obesity (e.g., endocrinologist, nutritionist, dietician, therapist, psychologist, etc.) or hospitalisation (e.g., a third or more 3-week BWRP) represented the possible options of medical supervision.

The main finding of the present study was the association of weight loss, detected one year after (i.e., before BWRP at T2) with medical supervision. According to our statistical analysis, only one session of medical supervision was sufficient to cause more than a 1-point or 0.1-point decrease in BWRP-induced weight loss, defined as ΔBMI or ΔBMI-SDS, respectively.

This remarkable finding was further documented by the more frequent medical supervision in responsive subjects, i.e., children and adolescents with obesity having a weight loss (ΔBMI) <−5 kg/m^2^, which represents a very stringent cut-off, when compared to the weight loss induced on average by our in-hospital 3-week BWRP (i.e., about −2 kg/m^2^ in terms of ΔBMI) [16,17,18].

The reasons of the positive carry-over effect exerted by medical supervision may be different.

For instance, one can rightly suppose an obese subject to be more psychologically motivated when medically supervised [19,20]. This encouragement by our healthcare personnel could be more effective in children and adolescents with obesity, who are reported to frequently drop out from interventions entailing dietetic, behavioural and life-style changes and easily gain body weight [3,21].

However, somebody could object to our view because our responsive group is likely to include the subjects who were more willing to lose body weight and that spontaneously required a more frequent medical supervision. This assumption, which is not negative in terms of healthcare, is not very convincing, because obese females, who are generally more willing to lose body weight [22], were, in the present study, as medically supervised as the male counterpart, with a similar weight loss. Furthermore, subjects having severe obesity (class 2 and 3) are generally those that are more (not only psychologically, but also socioculturally) discouraged/reluctant to undertake a long-term BWRP [23]. Our study contradicts this assertion because children and adolescents with severe obesity were those receiving a more frequent medical supervision.

Although patients’ participation is fundamental for achieving therapeutic success, we believe that the constant tailoring of any intervention to the specific clinical characteristics of the obese patients (e.g., the presence of comorbidities or psychological status) plays a pivotal role [24,25,26]. In particular, the medical and paramedical staff of our institution, through repeated sessions of medical supervision, can make therapeutic adjustments that, meeting a patient’s different needs, ensure the achievement of even ambitious targets in terms of weight loss and cardiometabolic outcomes [27].

It is noteworthy that, in the present study, weight loss was higher in children and adolescents with metabolic syndrome than those without, although medical supervision was quantitatively the same. Since we have demonstrated that the effectiveness of an (isolate) BWRP is similar in obese subjects with or without metabolic syndrome [17], this would suggest that the “qualitative” features of medical supervision administered to obese adolescents with metabolic syndrome represent a valuable therapeutic component for the success of any intervention in this group of patients who, by contrast with those with simple obesity, need more aggressive clinical management, including more careful medical supervision [28].

The beneficial effects of a more frequent medical supervision extended far beyond weight loss because, in the present study, we have also documented a parallel improvement of a set of auxometric (e.g., WC, FFM and FM) and cardiometabolic parameters (e.g., SBP, T-C, LDL-C, glucose and insulin) with the increasing weight loss, a result that, although expected, was particularly evident in the responsive group, characterised by numerous sessions of medical supervision. Among others, the most relevant cardiometabolic parameter that decreased during the 1-year follow-up was the change in IDF criteria for the diagnosis of metabolic syndrome. As use of antihypertensive, hypolipidemic and hypoglycaemic drugs was limited in our population of children and adolescents with obesity (both at T1 and T2), improvement of cardiometabolic parameters should be mainly attributed to the weight loss itself rather than to the drug prescriptions. Thus, the real-world evidence of the effectiveness of (our) medical supervision is likely to rest on repeated BWRPs and/or persuasive changes in life-styles (e.g., diet and physical activity), with no (or at least few) changes in drug prescription, an option of our clinical practice preferentially reserved to obese adults rather than children/adolescents [29].

Before closing, some limitations of our study should be mentioned.

One year follow-up may be a short period. As obesity is a chronic disease that requires a long-lasting multidisciplinary treatment [30], future studies should be planned to evaluate the effectiveness and, importantly, the persistence of medical supervision over a longer time period.

Due to the real-world setting of the study, we do not know the predominance of physician’s recommended vs. patient’s requested medical supervision. Anyway, to incentivise medical supervision in the real-world fight against obesity, the formation of qualified medical and paramedical personnel, working in an integrated multidisciplinary team, and the education of patients who should be aware that obesity is not an aesthetic problem but a chronic disease associated with even life-threatening comorbidities, might be valuable objectives of future investments in public health [27,31]. Educational programs should be early promoted in primary/secondary schools, being the population of interest that of children and adolescents [32].

We have focused our interest on medical supervision, but we cannot rule out the existence of other factors that, in synergy with or in opposition to medical supervision, might have had a role in determining the response to the initial BWRP during the 1-year follow-up. For instance, the pressure exerted by young patients’ parents having a different education level and socioeconomic status [33,34], the patients’ clinical history preceding the initial BWRP [35], or a patient’s access to healthcare services different from those supplied by our institution represent missing information.

Finally, we are aware that that visits free of charge improve the outcomes of a BWRP [36]. However, the out-patient clinic visits were also cost-free (i.e., covered by the National Health System). Thus, the economic difficulties cannot be considered as a possible interferent factor in determining the number of visits during the study period.

## 5. Conclusions

Based on the results of a real-world experience carried out in a population of children and adolescents with obesity, who had undergone an initial BWRP and were followed up for 1 year, frequent medical supervision increases the subsequent weight loss, with a parallel improvement of a set of auxometric and cardiometabolic parameters. Prospectively, incentivising regular medical supervision would mean reducing the risk of BWRP failure and body weight gain and, importantly, counteracting the detrimental transition from simple obesity to metabolic syndrome, an event that, in the pediatric context, is particularly critical [37]. Socio-political strategies should be adopted to promote not only access to an effective BWRP, but also long-term medical supervision to maintain or further increase the rapid (3-week) BWRP-induced weight loss.

## Figures and Tables

**Table 1:** Linear regression of changes in auxometric and metabolic parameters in the entire population of children and adolescents with obesity, being ΔBMI the independent variable.

Parameter	Mean ± SD	r^2^	Intercept	Coefficient	*p*
WC (cm)	−4.2 ± 12.3	0.3645	−0.9109	1.912	<0.0001
HC (cm)	−1.1 ± 8.5	0.6593	1.961	1.777	<0.0001
WHR	0.0 ± 0.1	0.00587	−0.02338	0.001526	0.3402
SBP (mmHg)	−1.2 ± 12.7	0.03249	−0.2355	0.59	0.0239
DBP (mmHg)	−0.4 ± 9.3	0.01242	0.03861	0.2653	0.1646
Total Cholesterol (mg/dL)	−5.9 ± 21.3	0.08632	−3.209	1.606	0.0002
HDL (mg/dL)	0.9 ± 7.2	0.01678	0.4679	−0.241	0.1058
LDL (mg/dL)	−6.1 ± 23.1	0.07851	−3.264	1.66	0.00004
Triglycerides (mg/dL)	−8.0 ± 34.0	0.08301	−3.737	2.518	0.0003
Glucose (mg/dL)	−2.6 ± 8.9	0.09049	−1.244	0.4541	0.0001
Insulin (mIU/L)	−9.1 ± 15.7	0.2439	−5.502	1.856	<0.0001
HOMA-IR	−2.1 ± 3.5	0.2538	−1.232	0.4249	<0.0001
FM (kg)	−1.4 ± 9.7	0.7625	2.347	2.171	<0.0001
FFM (%)	0.2 ± 6.4	0.3505	−1.491	−0.9726	<0.0001
IDF Criteria (n)	−0.1 ± 1.0	0.1276	0.0157	0.08761	<0.0001

Note: the change (i.e., Δ) for dependent/independent variables refers to the difference between the basal values of the parameter at T1 and T2, being T1 or T2 the time of the first or second hospitalisation for the 3-week BWRP, respectively. See the text for further details. Abbreviations: BMI, body mass index; DBP, diastolic blood pressure; FFM, fat free mass; FM, fat mass; HC, hip circumference; HDL, high density lipoprotein; HOMA-IR, homeostatic model assessment for insulin resistance; IDF, International Diabetes Federation; LDL, low density lipoprotein; SBP, systolic blood pressure; SD, standard deviation; wC, waist circumference; WHR, waist to hip ratio.

**Table 2:** Changes in auxometric and metabolic parameters in children and adolescents with obesity, defined as responsive, stable or non-responsive in accordance with ΔBMI (i.e., <−5, >−5 and <5 or >5 kg/m^2^, respectively).

Parameter	Responsive	Stable	Non-Responsive
BMI (kg/m^2^)	−7.1 ± 1.9 ^a,b^	−0.9 ± 2.6 ^a^	6.8 ± 1.8
BMI-SDS	−0.7 ± 0.3 ^a,b^	−0.2 ± 04 ^a^	0.6 ± 0.4
WC (cm)	−11.7 ± 10.8 ^a,b^	−3.1 ± 11.8 ^a^	8.3 ± 12.4
HC (cm)	−9.8 ± 6.6 ^a,b^	0.2 ± 7.2 ^a^	12.4 ± 4.5
WHR	0.0 ± 0.1	0.0 ± 0.1	0.0 ± 0.1
SBP (mmHg)	−3.4 ± 12.7	−0.9 ± 12.9	1.4 ± 10.7
DBP (mmHg)	−1.9 ± 7.4	0.0 ± 9.9	−0.7 ± 4.5
Total Cholesterol (mg/dL)	−14.2 ± 22.3	−4.6 ± 21.0	4.9 ± 10.3
HDL (mg/dL)	3.8 ± 5.7 ^a,b^	0.5 ± 7.4	−4.7 ± 6.6
LDL (mg/dL)	−16.2 ± 23.3 ^a,b^	−4.3 ± 22.8	5.6 ± 14.5
Triglycerides (mg/dL)	−21.4 ± 33.7 ^a^	−6.6 ± 33.0	22.6 ± 32.1
Glucose (mg/dL)	−5.0 ± 4.5 ^a,b^	−1.6 ± 5.4	−8.1 ± 33.9
Insulin (mIU/L)	−16.8 ± 12.2 ^a,b^	−7.7 ± 14.4 ^a^	−2.5 ± 32.6
HOMA-IR	−3.8 ± 2.7 ^a,b^	−1.8 ± 3.2 ^a^	−0.1 ± 7.2
FM (kg)	−13.1 ± 5.4 ^a,b^	0.6 ± 8.0 ^a^	14.1 ± 2.9
FFM (%)	5.2 ± 4.1 ^a,b^	−0.7 ± 6.2 ^a^	−6.4 ± 5.9
IDF Criteria (n)	−0.7 ± 1.0 ^a,b^	0.0 ± 0.9	0.4 ± 1.2

Note: the change (i.e., Δ) for dependent/independent variables refers to the difference between the basal values of the parameter at T1 and T2, being T1 or T2 the time of the first or second hospitalisation for the 3-week BWRP, respectively. See the text for further details. Abbreviations: BMI, body mass index; DBP, diastolic blood pressure; FFM, fat free mass; FM, fat mass; HC, hip circumference; HDL, high density lipoprotein; HOMA-IR, homeostatic model assessment for insulin resistance; IDF, International Diabetes Federation; LDL, low density lipoprotein; SBP, systolic blood pressure; SD, standard deviation; SDS, standard deviation score; wC, waist circumference; WHR, waist to hip ratio. ^a^ *p* < 0.05 vs. non-responsive group. ^b^ *p* < 0.05 vs. stable group.

## Data Availability

The datasets used and/or analyzed in the present study are available from the corresponding author on reasonable request.

## Abbreviations

BMI: body mass index; CV, coefficient of variation; DBP, diastolic blood pressure; FFM, fat free mass; FM, fat mass; HC, hip circumference; HDL, high density lipoprotein; HOMA-IR, homeostatic model assessment for insulin resistance; CI, confidence interval; IDF, International Diabetes Federation; LDL, low density lipoprotein; SBP, systolic blood pressure; SD, standard deviation; SDS, standard deviation score; wC, waist circumference; WHC, World Health Organisation; WHR, waist to hip ratio.
